# Effect of noisy galvanic vestibular stimulation on dynamic posture sway under visual deprivation in patients with bilateral vestibular hypofunction

**DOI:** 10.1038/s41598-021-83206-z

**Published:** 2021-02-19

**Authors:** Po-Yin Chen, Ying-Chun Jheng, Chien-Chih Wang, Shih-En Huang, Ting-Hua Yang, Po-Cheng Hsu, Chia-Hua Kuo, Yi-Ying Lin, Wei-Yi Lai, Chung-Lan Kao

**Affiliations:** 1 Department of Physical Therapy and Assistive Technology, National Yang-Ming Chiao-Tung University, Taipei, 11221 Taiwan; 2 Department of Physical Medicine and Rehabilitation, Taipei Veterans General Hospital, Taipei, 11217 Taiwan; 3 School of Medicine, National Yang-Ming Chiao-Tung University, Taipei, 11221 Taiwan; 4 Department of Physical Medicine and Rehabilitation, Taipei Veterans General Hospital Yuli Branch, Hualien, 98142 Taiwan; 5 Department of Otolaryngology, College of Medicine, National Taiwan University, Taipei, 106216 Taiwan; 6 Physical Medicine and Rehabilitation, National Taiwan University Hospital, Bei-Hu Branch, Taipei, 10845 Taiwan; 7 Department of Sports Sciences, University of Taipei, Taipei, 11153 Taiwan; 8 Department of Medical Research, Taipei Veterans General Hospital, Taipei, 11217 Taiwan; 9 Center for Intelligent Drug Systems and Smart Bio-Devices (IDS2B), National Yang-Ming Chiao-Tung University, Hsinchu, 30093 Taiwan; 10 Institute of Clinical Medicine, National Yang-Ming Chiao-Tung University, Taipei, 11221 Taiwan

**Keywords:** Outcomes research, Motor control, Sensorimotor processing

## Abstract

A single-blind study to investigate the effects of noisy galvanic vestibular stimulation (nGVS) in straight walking and 2 Hz head yaw walking for healthy and bilateral vestibular hypofunction (BVH) participants in light and dark conditions. The optimal stimulation intensity for each participant was determined by calculating standing stability on a force plate while randomly applying six graded nGVS intensities (0–1000 µA). The chest–pelvic (C/P) ratio and lateral deviation of the center of mass (COM) were measured by motion capture during straight and 2 Hz head yaw walking in light and dark conditions. Participants were blinded to nGVS served randomly and imperceivably. Ten BVH patients and 16 healthy participants completed all trials. In the light condition, the COM lateral deviation significantly decreased only in straight walking (*p* = 0.037) with nGVS for the BVH. In the dark condition, both healthy (*p* = 0.026) and BVH (*p* = 0.017) exhibited decreased lateral deviation during nGVS. The C/P ratio decreased significantly in BVH for 2 Hz head yaw walking with nGVS (*p* = 0.005) in light conditions. This study demonstrated that nGVS effectively reduced walking deviations, especially in visual deprived condition for the BVH. Applying nGVS with different head rotation frequencies and light exposure levels may accelerate the rehabilitation process for patients with BVH.

**Clinical Trial Registration** This clinical trial was prospectively registered at www.clinicaltrials.gov with the Unique identifier: NCT03554941. Date of registration: (13/06/2018).

## Introduction

Walking is not only the most important type of locomotion performed by humans in daily activities but is also a multisensory process including optic information (visual), body information (proprioceptive), and the head position (vestibular). During locomotion, the vestibular function crucially contributes to static and dynamic balance control. The vestibular organs send information regarding the position of, and changes in, the linear and angular velocities of the head to the cerebrum and cerebellum, which controls gaze, equilibrium, and muscle tone^1,2^. Vestibular input and proprioception provide important information about spatial perception that is important for reflexes, orientation, and spatial coordination^3,4^. Bilateral vestibular hypofunction (BVH) causes continuous unsteady posture and blurred vision during head movements, leading to imbalance and falls. Injuries caused by falls lead to high social and economic burdens.

BVH patients have impaired dynamic visual acuity; oscillopsia occurs during head or body movements. Studies have suggested that head movements are crucial for vestibular rehabilitation^5,6^. Because of decreased or absent vestibular input, patients become more reliant on sensory modalities such as vision and proprioception. When BVH patients walk on uneven surfaces in the dark, their risk of falls increases when they rely on deficit of the vestibular system^7–9^.

Galvanic vestibular stimulation (GVS) is a non-invasive technique that decreases the firing threshold of vestibular nerves. The mechanism involves electrical stimulations being sent to vestibular nerves through an electrode placed on the mastoid area bilaterally^10–12^. By applying a direct current to the mastoid process with the bipolar GVS, the posture starts to sway towards the anode because the participant perceived the feeling of rotation^13^. Recently, this intervention has provided insights into balance control, including balance re-education in physical therapy. Recent studies have used noisy galvanic vestibular stimulation (nGVS) to facilitate control of standing balance and perceptive spatial functions^15–18^. Using an imperceptible GVS noise level, sway during quiet standing decreased in both healthy participants and BVH patients^15,19^. In addition, Serrador et al. revealed enhancing vestibular ocular function by imperceptible GVS noise^14^.

In previous nGVS studies of the vestibular system, researchers conducted pre-tests to define the optimal intensity of nGVS. By measuring the center of mass (COM) trajectories of participants while standing under nGVS conditions with different imperceptible levels of intensity, investigators identified one participant-specific current to yield the most stable performance. Each participant then participated in balance and locomotion experiments at his/her optimal intensity. Iwasaki et al. investigated the effects of randomized nGVS during general walking with inertial measurement units (IMU)^20^, and Wuehr et al. and Mulavara et al. measured the effects during treadmill walking^21,22^. All these studies found that nGVS improved gait performance. However, all the tests used in these studies were conducted in relatively light environments, with each standing/walking trial being performed at a constant speed on a treadmill. However, individuals may increase their dependence on the vestibular system and proprioception in the dark due to sensory reweighting. Furthermore, when walking on a treadmill, the participant may lack optic flow and slightly guide their steps by the walking belt of the treadmill. These experimental conditions did not mimic real-life scenarios. Most importantly, the head liner and angular velocities trigger the vestibular afferents that cause oscillopsia. In addition, for position reconstruction, optical motion capture can physically see each marker and detect the slightest of movements with better accuracy than other types of motion capture. Therefore, in the present study, using a three-dimensional (3D) optical motion capture system of high accuracy (system error in dynamic conditions (8 cameras, 100 Hz) is less than 2 mm), we tested the effects of nGVS on deviations during walking with the head facing forward or rotating at 2 Hz in both light and dark conditions in healthy and BVH participants. We hypothesized that individuals would reduce the deviations during various dynamic conditions with optimal nGVS, mainly in the dark. We aimed to explore the different effects of nGVS when individuals walk with or without head motion and light to identify the optimal rehabilitation protocol for BVH.

## Results

**Figure 1:**
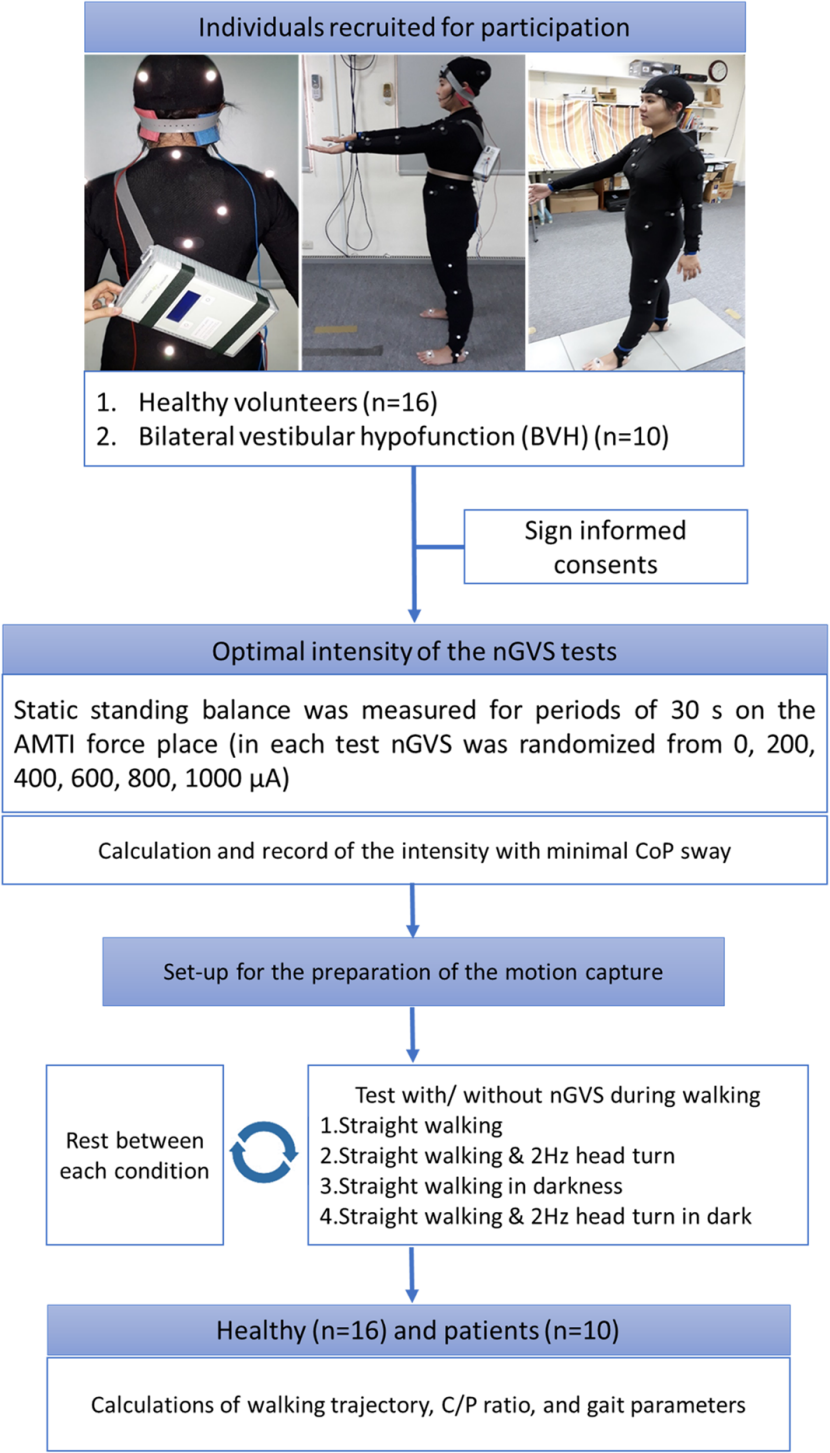
Flow chart of all the tests with participant’s preparation. Participants wore a motion capture (mocap) suit with plug-in-gait marker setting. A galvanic vestibular stimulation (nGVS) device was fixed on participant’s back firmly. *nGVS* noisy galvanic vestibular stimulation, *CoP* center of pressure, and *C/P ratio* chest–pelvis ratio.

We recruited ten BVH patients (one man/nine women; mean age 51.3 ± 18.6 years) and 16 healthy participants (eight men/eight women; mean age 40.9 ± 14.2 years) (Fig. 1). The demographic and etiological data for the patients and conditions are shown in Table 1.

**Table 1:** Demographic data for patients with BVH.

	Sex/age	R caloric (°/s)	L caloric (°/s)	Diagnosis	Onset time (m)	Training (m)	Optimal nGVS (µA)
P1	F/64	7	8	idiopathic BVH	2.5	NA	200
P2	F/63	9	8	idiopathic BVH	1.5	NA	800
P3	F/41	4	8	idiopathic BVH	2	1	600
P4	F/68	8	6	idiopathic BVH	2	1	200
P5	F/67	9	9	idiopathic BVH	1	1	200
P6	F/49	1	6	idiopathic BVH	2.5	0.5	600
P7	M/26	0	9	idiopathic BVH	2	NA	600
P8	F/73	6	8	idiopathic BVH	2	NA	200
P9	F/40	7	7	idiopathic BVH	1	NA	200
P10	F/22	8	9	idiopathic BVH	1.5	NA	1000

*F* female, *M* male, *BVH* bilateral vestibular hypofunction, *m* month, *nGVS* noisy galvanic vestibular stimulation.

### Effects of nGVS on gait performance in BVH patients in terms of walking deviations, the C/P ratio and gait parameters

#### Primary outcome

In BVH patients, nGVS decreased the deviation during walking by approximately 25–35% compared with patients without GVS (Table 2). Walking deviation reflects the participant’s level of balance control during locomotion. nGVS reduced the deviations during straight walking in the patients, regardless of whether the environment was lit without head rotation (condition 1) or dark without head rotation (condition 3). The walking deviation in light without head rotation was 3.02 ± 1.48% without nGVS and 2.39 ± 0.95% with nGVS (*p* = 0.037); that of walking deviation in the dark without head rotation was 4.28 ± 1.90% without nGVS and 2.80 ± 1.02% with nGVS (*p* = 0.017). When patients walked with head yaw rotations at 2 Hz, the walking deviation significantly improved only in the dark (6.40 ± 3.20% without nGVS and 4.06 ± 2.50% with nGVS; *p* = 0.047). Under the light condition, there was no significant effect of nGVS on the change in walking deviation in the patient group during head yaw rotations at 2 Hz.

**Table 2:** Walking deviation during four conditions.

Conditions	Control (%)	SD (control)	nGVS (%)	SD(nGVS)	Z test	*p* value
**Patients group**						
Straight walking (1)	3.02	1.48	2.39	0.95	− 2.090	0.037*
Straight walking, 2 Hz (2)	3.63	0.96	3.12	0.89	− 1.376	0.169
Straight walking, Darkness (3)	4.28	1.90	2.80	1.02	− 2.395	0.017*
Straight walking, 2 Hz, Darkness (4)	6.40	3.20	4.06	2.50	− 1.988	0.047*
**Healthy group**						
Straight walking (1)	3.16	1.72	2.97	1.76	− 0.465	0.642
Straight walking, 2 Hz (2)	3.72	1.09	3.10	1.14	− 2.172	0.030*
Straight walking, Darkness (3)	3.24	1.61	2.22	0.71	− 2.223	0.026*
Straight walking, 2 Hz, Darkness (4)	3.61	1.70	2.78	2.50	− 2.120	0.034*

This table show performances of participants in straight walking without or with head rotation, light, and nGVS (four conditions). The straight walking without head rotation in the light is condition 1 and in the dark is condition 3; walking and make yaw head rotations at a frequency of 2 Hz the light is condition 2 and in the dark is condition 4.

*Contro* without nGVS, *nGVS* noisy galvanic vestibular stimulation, *Z* Z value of Wilcoxon signed ranks test, *SD* standard deviations.

*p < 0.05.

#### Secondary outcomes

##### Chest–pelvic (C/P) ratio

**Figure 2:**
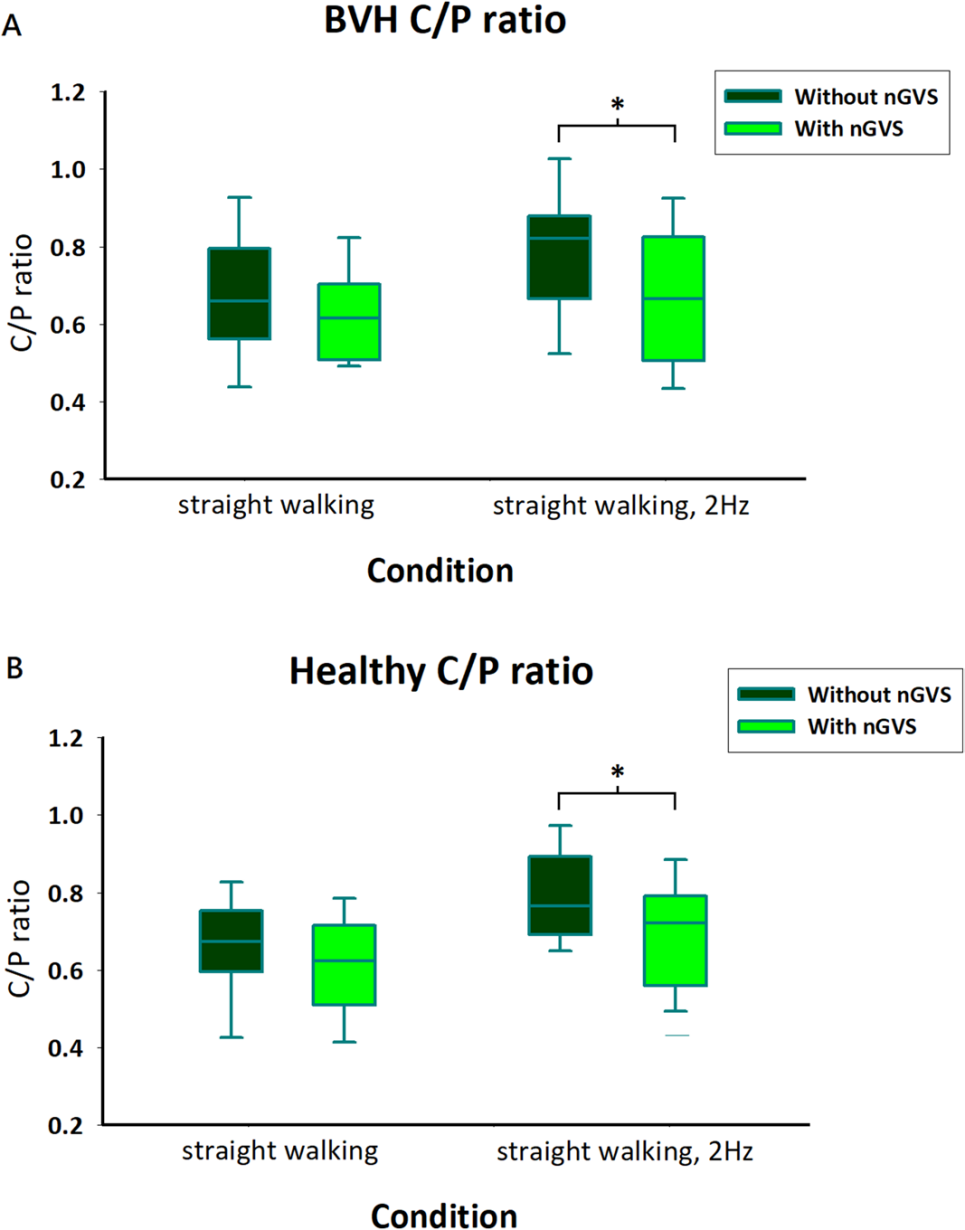
C/P ratio of patients with BVH (**A**) and healthy participants (**B**) in the light condition. Only condition 2 showed reduced deviations during noisy galvanic vestibular stimulation (nGVS). *BVH* bilateral vestibular hypofunction, *C/P ratio* chest–pelvis ratio, *p < 0.05.

To further evaluate the effect of nGVS on postural stability in BVH patients, we investigated changes in postural control and gait. The C/P ratio revealed the coordination and stiffness of trunk postural control. When walking in the light condition with 2 Hz head rotations, the patients exhibited a lower C/P ratio with nGVS than without nGVS, indicating a less stiff walking pattern. The C/P ratio decreased with nGVS (0.78 ± 0.16 to 0.68 ± 0.17; *p* < 0.01; Fig. 2A).

##### Gait parameters

**Figure 3:**
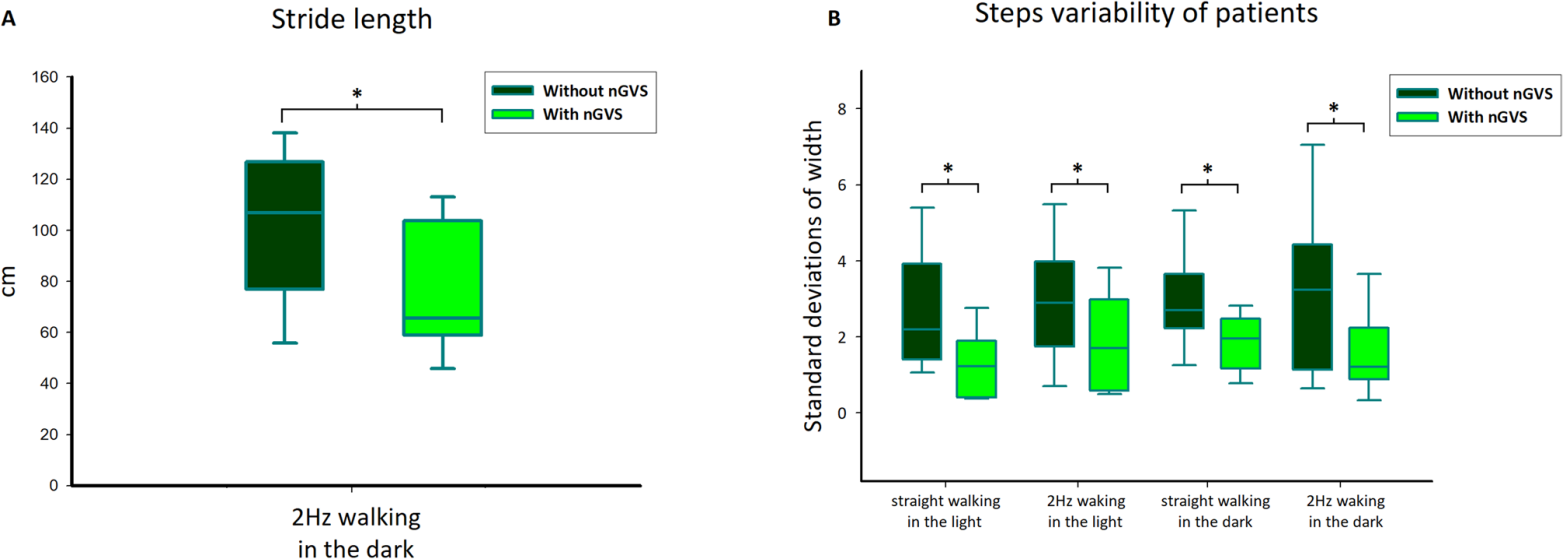
Comparisons of stride length (**A**) and steps variability (**B**) in patients. Patients showed reduced step length in light condition and steps variability in all conditions during noisy galvanic vestibular stimulation (nGVS), *p < 0.05.

For gait performance, stride length decreased with nGVS while walking only in the dark condition with head rotations (condition 4) (*p* < 0.22) (Fig. 3A). The standard deviation of the step width decreased in all conditions with nGVS (Fig. 3B; Table 3).

**Table 3:** The stride width variability of patients during four conditions.

Conditions	Control	SD (control)	nGVS	SD (nGVS)	Z test	*p* value
**Step width standard deviations**						
Straight walking (1)	2.64	1.52	1.29	0.86	− 2.599	0.009*
Straight walking, 2 Hz (2)	2.90	1.56	1.83	1.28	− 2.191	0.028*
Straight walking, darkness (3)	2.98	1.19	1.82	0.75	− 2.191	0.028*
Straight walking, 2 Hz, darkness (4)	3.26	2.02	1.54	1.05	− 2.293	0.022*

*Control* without noisy galvanic vestibular stimulation, *nGVS* noisy galvanic vestibular stimulation, *SD* standard deviations.

*p < 0.05.

### Effects of nGVS on gait performance in healthy participants in terms of walking deviatoins and the chest–pelvis (C/P) ratio

#### Primary outcome

In healthy participants, the walking deviation decreased with nGVS in the task in the light with head yaw rotation (condition 2), conditions in the dark without (condition 3) and with head rotation (condition 4) (Table 2). The healthy participants had lower walking deviations when walking with head rotations and nGVS than in the other conditions, regardless of whether the environment was light or dark. The walking deviation in the task in the light with head yaw rotation (condition 2) was 3.72 ± 1.09% without nGVS and 3.10 ± 1.14% with nGVS (*p* = 0.01), and that in the task in the dark with head yaw rotation (condition 4) was 3.69 ± 1.70% without nGVS and 2.77 ± 2.50% with nGVS (*p* = 0.034). nGVS also decreased the deviation during straight walking in the dark [condition 3, 3.24 ± 1.61% without nGVS and 2.21 ± 0.71% with nGVS (*p* = 0.026)]. However, it did not affect the deviation during straight walking in the light, which is the typical walking condition.

#### Secondary outcome

The C/P ratio was lower with nGVS than without nGVS in the light while walking with head rotations in the healthy participants (condition 2, 0.80 ± 0.12 to 0.70 ± 0.14; *p* < 0.01, Fig. 2B).

## Discussion

By applying the optimal nGVS intensity, we provided evidence that the path deviation can be improved in both healthy and BVH participants, particularly when patients are walking with head yaw rotations at 2 Hz in the dark. Even though visual deprivation can be detrimental to walking performance, especially for BVH patients, our results showed that walking instability in the dark could be ameliorated in BVH patients. This is the first study addressing the effects of nGVS under visual deprivation conditions to the best of our knowledge. The C/P ratio improved during walking with yaw head rotations at 2 Hz only in the light condition in the BVH patients, and the mean magnitude of improvement was similar to the one observed in healthy participants. Our work also provides highly accurate measurements of gait parameters calculated using a 3D motion analysis system; nGVS decreased step width variability in both light and dark conditions.

BVH patients commonly present larger COM sway during stance, and this phenomenon can be effectively alleviated by applying nGVS on either side of their mastoid processes^15,19^. In the current study, BVH patients also showed larger walking trajectory deviations when walking without nGVS, suggesting instability during locomotion. This instability during walking may be attributed to impaired vestibular systems that cannot provide sensitive correct information about head movements. Regarding vestibular function, previous research has shown that GVS can increase the amplitude of vestibular evoked myogenic potentials^23^. Additionally, Hilliand et al. had found that nGVS modulates spatial memory in healthy young adults^24^. We found that vestibular function improvements during the dynamic task were possibly caused by the stochastic resonance of nGVS. The vestibular detection threshold may be lowered via stochastic resonance of nGVS on non-linear vestibular signals, and participants would detect and process subthreshold vestibular signals easily^25,26^. Therefore, nGVS decreased the walking deviation of the participants during our tests.

**Figure 4:**
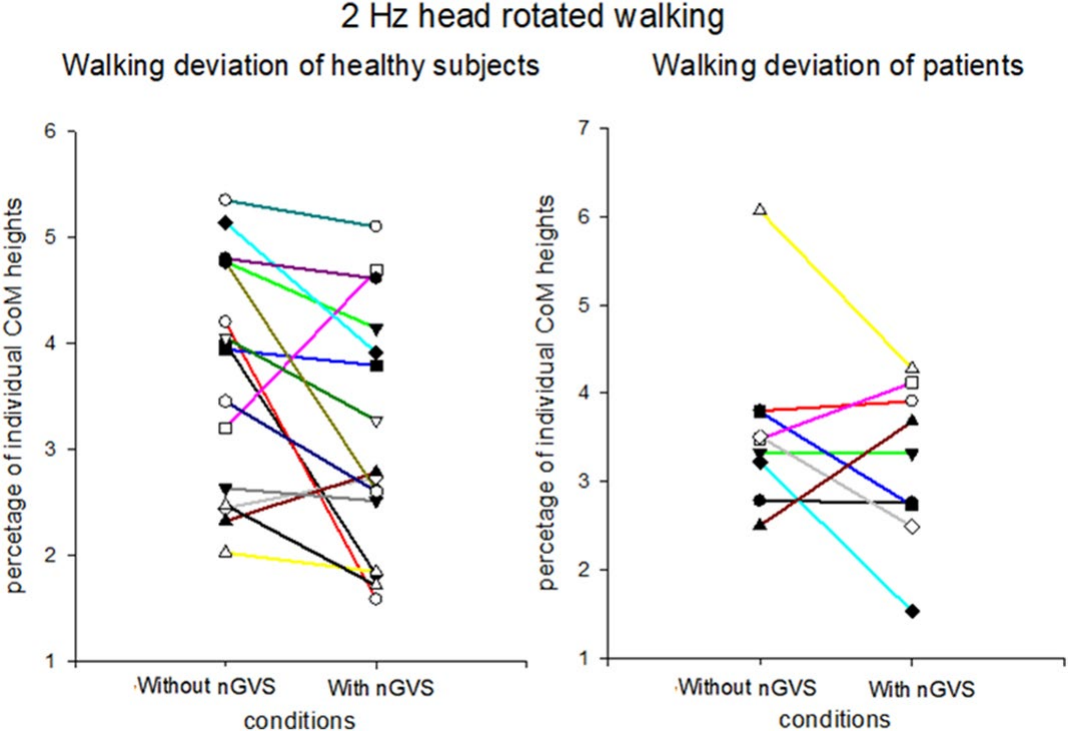
Comparison of walking deviations between healthy participants and patients. This figure shows the change of walking deviations by noisy galvanic vestibular stimulation (nGVS) during walking with 2 Hz head rotation. The results show the change of each healthy participant (left side) and patient (right side).

Nevertheless, the participants’ walking deviation did not decrease in all tests with nGVS. The healthy participants showed no changes in walking deviation during straight walking with nGVS. One possible reason is that straight walking did not exert a sufficient head angular velocity to cause path deviation. In contrast to healthy participants, the patients showed improved walking deviation values in straight walking, which may be indirect evidence of the roof effect in healthy participants during straight walking. Interestingly, the patient group showed significant improvements in walking in all conditions except for condition 2, i.e., walking with head yaw rotations at 2 Hz in the light (Fig. 4). We propose one possible explanation: the complex task, which triggers both the vestibulo-ocular and vestibulo-spinal reflexes in condition 2, is too difficult for the patients. Therefore, the effects of nGVS might have been masked by this complexity. This explanation is supported by our observation that patients had a relatively wider step width when walking with head turns than when walking without head turns, reflecting the possibility that it was more difficult for patients to adapt to vestibular perturbations in condition 2 than in condition 1.

In contrast to the findings of Iwasaki et al., we did not observe significant changes in stride length in the condition of walking in the light with either the head straight or rotating at 2 Hz in the yaw plane. Iwasaki et al. observed that stride length increased in patients when nGVS was applied^20^. We found that stride length decreased only when patients walked in the dark with head rotations (Fig. 3). This inconsistency may be attributed to the differences in the designs of these two studies. Iwasaki et al. determined the optimal stimulation intensity based on the participants’ step length increase during walking over 10–15 m. Our study used a similar method to find the optimal intensity of stimulation, as described by Wuehr et al.^21^, and allowed participants to walk over 4–5 m. Although we did not observe a difference in step length variability, we indeed found that the standard deviation of the step width (cm) decreased after nGVS was applied. Step width is the lateral distance between the left and right feet that can be affected by a sensory impairment or aging^27–29^. We found that the standard deviation of the step width decreased in the BVH patients, suggesting that nGVS increases gait stability. Previous studies showed that GVS affects foot placement during walking^30,31^. By correcting vestibular inputs via nGVS, vestibulospinal tract function may, in turn, be fine-tuned, allowing patients to have better control of their foot placement, leading to greater stability during walking.

Trunk control or coordination has rarely been discussed in previous studies. We showed that both healthy and patients had decreased C/P ratios when walking in the light with 2 Hz head yaw rotations. The decreased C/P ratio indicates an improvement in trunk coordination, i.e., the chest and pelvis turned in opposite directions in an out-of-phase rhythm. The phenomenon in which individuals do not exhibit out-of-phase trunk coordination during walking has been shown in various patient populations, including patients with stroke^32^, low back pain^33^, and Parkinson's disease^34^. Some researchers have observed that patients with impaired vestibular functions tend to have rigid trunk movements or become “en bloc”^1,35,36^. Spatial orientation tends to be impaired in vestibular patients because of a lack of accurate sensory afferents. Therefore, using this en bloc strategy, patients can control their head positions more easily based on the orientation of their trunks.

Wuehr et al. used IMUs to measure head and trunk motions in patients with their eyes closed when walking and found differences in the head and trunk movements with and without nGVS^21^. This finding is consistent with the C/P ratio results reported in our study for the dark condition. One possible explanation is that the sense of vision helps individuals determine their body segment orientations. Visual deprivation may reduce the capacity for body segment orientation control. Participants may adopt the en bloc strategy to control their trunk during dynamic or complex activities. Regarding cortical modulation, some researchers found that the parieto-insular vestibular cortex (PIVC) contains multisensory convergence information of self-motion cues with external visual object motion^37,38^. The PIVC receives primary vestibular inputs, and many of its neuron activities correlate with proprioception^37^ and the task of visual tracking^38^. Therefore, tasks with or without visual input may affect the processing of the vestibular input at the level of the central nervous system in the PIVC. With nGVS, vestibular functions within the vestibular apparatus are activated, leading to a more profound influence on the C/P ratio in light conditions—in which participants receive more visual sensory input and can exploit and integrate multiple sensory information during walking—than in darkness.

Visual-vestibular interactions have been shown to play a fundamental role in maintaining trunk balance^39^. Reciprocal inhibition failure between visual and vestibular cortex interactions has been proposed as an important factor contributing to the imbalance in brain activity and thus vestibular hypofunction in BVH patients^40^. Increases in visual activity have been shown to correlate with perceived dizziness^41^, suggesting that BVH patients largely rely on vision. This finding is consistent with that of our study, as BVH patients revealed significantly increased walking deviation in the dark than in the light when a visual function was deprived. The deviation increased significantly after head rotations were added in the light than in the dark, suggesting the essential role of the cerebellum-vestibular pathway in the visual deprivation condition.

This result suggests that nGVS has a neuromodulatory effect and can either compensate for the original pathway or re-modulate other possible brain areas to facilitate the other cortical areas in the visual deprivation condition. Kwan et al. also revealed the neural substrate underlying GVS-evoked perceptual in monkeys^42^. The vestibular nerve projection through the thalamocortical pathway has also been shown to be directly affected by nGVS. This nGVS modulatory effect was associated with not only enhanced motor task performance but also attention and mental workload^43–47^. Increased mental function and motor control may have a large influence on maintaining posture sway in visual deprivation conditions.

Another potential mechanism may be that nGVS downregulates the activity of the primary visual cortex, thereby suppressing visual-vestibular symptoms^40^. In an fMRI study, nGVS changed brain activity in areas of the cerebellum and visual cortex associated with dizziness-related impairment, reflecting that neural plasticity may improve impaired self-motion perception^48^.

In the study, the effects of nGVS were observed in real-time. Lacking the measurements of post-stimulation behavioral responses is our major limitation. In our future work, we will include more participants of different ages and increase walking distance in the trials to study nGVS effects on prolonged walking trials. Post-stimulation effects will be investigated to determine whether there is long-term potentiation.

The outcome in the current study may provide a potential for positive effects on dynamic balance and functional training in clinical rehabilitation. Clinical therapists may integrate the effect of nGVS with treatment to allowt BVH patients to begin their physical therapy early. Patients also could increase the complexity of clinical balance training to accelerate adaptation and compensation. In addition, for patients with poor compensation, the device with optimal stimulation might be the potential prosthesis for walking or daily activities.

Using optic motion capture analysis, we provided evidence that nGVS improves dynamic posture stability both in healthy participants and BVH patients. We also found that rotating the head during walking may be too complex for patients. However, in this multiple sensory condition, patients showed improved chest–pelvis coordination in the light condition with head rotations after receiving nGVS. Our findings suggest that integrating nGVS with vestibular rehabilitation can accelerate the progress of BVH participants. The elucidation of the underlying mechanisms by which nGVS provides its benefits requires further brain imaging studies.

## Methods

### Study design

A single-blind, nonrandomized study was conducted to compare walking parameters with and without nGVS stimulation in both healthy participants and patients diagnosed with BVH. The participants were blinded to these test conditions, whether with or without imperceptible nGVS.

### Participants, clinicians, and centers

Participants who met the selection criteria were recruited through primary hospital referrals. The primary investigator was a registered physiatrist at Taipei Veterans General Hospital with 20 years of experience treating vestibular patients. Gait analysis was performed by a physiotherapist with 10 years of gait analysis experience. The inclusion criteria were as follows: (1) ability to walk for 10 min in one trial; (2) absence of a history of orthopedic surgeries or other neurological diseases; and (3) sufficient cognition and motivation to participate. The BVH were diagnosed based on self-reported histories and the results of head thrust tests, horizontal and vertical head-shaking nystagmus tests, and bi-thermal caloric irrigation with 60 s air (AIRSTAR, Micromedical Technologies, IL, USA) using the following criteria: total response of slow phase velocity < 20°/s [R(right) warm + R cold + L(left) warm + L cold)] at both ears.

### Interventions

#### Noisy galvanic vestibular stimulation setting

nGVS was delivered using a DC-STIMULATOR PLUS (Eldith, NeuroConn GmbH, Ilmenau, Germany), and 5 cm × 5.5 cm electrodes were centered over both mastoid processes of the participants. The participants wore tight suits, and a standard plug-in-gait model set was used with the motion capture system and forceplate.

### Procedures

We used zero-meaned the white noise GVS signal, which ranged from 0.02 to 10 Hz. The peak amplitudes of the GVS signal were 0, 200, 400, 600, 800, and 1000 µA in all participants. These intensities were applied in random order. The rest time was set to 2 min between each measurement and 3 min between zero amplitude and stimulation conditions (200, 400, 600, 800, and 1000 µA) to prevent after-effects of the stimulation from confounding the results (Fig. 1).

The optimal intensity for each participant and patient was determined based on the center of pressure (COP) sway during the standing test. The participants stood on a force plate with a sampling rate of 100 Hz (40 cm × 40 cm, AMTI, USA), with their eyes open and feet together. The data from the force plate were collected by motion analysis software with a lowpass filter and a cut-off frequency of 6 Hz. We calculated the root mean square (RMS) of the mean COP sway at every time point from each trial. We then compared the RMS of the COP to determine the minimal value, corresponding to the optimal intensity with minimal sway. COP was measured during the interventions (30 s), and both the fade-in and fade-out times were set to 8 s. COP was measured for 30 s after the fade-in period.

### Locomotion experiments using the motion capture system

We conducted a locomotion test using a motion capture system with a sampling rate of 100 Hz (VICON, Oxford, UK) for motion analysis. Markers’ data were filtered with a 4th order lowpass Butterworth filter with a cut-off frequency of 6 Hz. The space used for the experiment measured 10 m × 5 m and was surrounded by eight infrared cameras (TX-20, 2 million pixels, Oxford, UK). The motion marker set used was based on the VICON plug-in gait model (five markers on the head, six markers on the chest, six markers on the pelvis, and 26 markers on limbs), and the markers measured 14 mm. We added one marker on the top of the head, one marker on the right scapula between the level of C7 and T1, and two markers on the iliac crest. Each participant participated in four walking tests at their preferred gait speed: straight walking without head rotation in the light (condition 1) and walking with yaw head rotations at 2 Hz in the light (condition 2); straight walking without head rotation in darkness (condition 3), and walking with yaw head rotations at 2 Hz in darkness (condition 4). Regarding the 2 Hz head yaw, participants were instructed by a metronome while walking. All conditions were executed with 0 µA nGVS and individual-specific optimal intensities. Participants were asked to walk straight from a fixed starting marker on the floor and stop at another marker, which was 5 m away in the forward direction. Then, researchers demonstrated the trial and instructed participants to practice until they understood certainly. Each participant performed each condition four times (twice without nGVS and twice with nGVS). Sham stimulation was not performed because the stimulation was gentle, and the participants could not feel the stimulation. The trials without nGVS were performed first to avoid possible stimulation after-effects.

### Outcome measures

The primary outcome was the body’s lateral deviation during the walking task. In all the conditions, the COM trajectories were collected from the start to the end of walking, and the airline distance was around 5 m. The lateral deviation of the COM along a path (straight line) was calculated by averaging the mean magnitude of the deviation (absolute difference from every sample) of each sample relative to a simulated straight line. To control for differences in leg length, we normalized the mean amplitude (cm) of the lateral deviation by the height (cm) of each participant’s COM. These data are expressed as percentages. A smaller percentage for an individual means a smaller sway and better balance control.

We applied nGVS with the participant-specific optimal intensities during all walking tests for approximately 10 s. We evaluated the real-time effect of nGVS on postural control during the dynamic task. Static balance was compared between at least one 30-s trial with the participant’s optimal intensity at 200–1000 µA and a trial with an intensity of 0 µA.

The secondary outcome measures were gait parameters, including gait speed, step length, step width, and the standard deviations of step length and step width, measured using the marker data and analyzed by the VICON software. For the chest–pelvis ratio (C/P ratio), we normalized the chest and pelvis data to the successive chest and pelvis rotation angles for each trial. The ratio was calculated by summing the chest and pelvis rotation angles in the yaw plane. The angles at each time point in the whole temporal sequence were then averaged to calculate the ratio. When a participant turned his or her chest and pelvis in the same direction during walking, using stiff control of the trunk, the chest and pelvis were considered in phase, and the C/P ratio was high. Conversely, a smaller C/P ratio indicated that the chest and pelvis were out of phase, which is the predominant way to regulate trunk stability during walking.

This study estimated sample size based on data published in the change of gait pattern by nGVS with the G*Power 3.1.9.7 software (Franz Faul, University Kiel, Germany). We used the improvement data in the base of support with nGVS compared to sham stimulation in a previous study^49^. Accordingly, we achieved 80% statistical power and α = 0.05 using at least N = 10 participants in each group.

### Statistical analysis

All statistical analyses were performed using IBM SPSS Statistics for Windows 20.0 statistical software (Released 2011. Armonk, NY: IBM Corp.), and the significance level was set at 0.05. The effects of nGVS were tested by comparisons and differences between performances without and with nGVS in each condition. Because of the small sample sizes, the Wilcoxon Signed Ranks test was used for the participants for the paired comparison.

### Ethics approval of research

This study was approved (authorization number: 2015–12-004C) by the institutional review board of Taiwan Taipei Veteran General Hospital as well as the Food and Drug Administration (Taiwan, ROC). All participants’ data was anonymized by a study number that did not contain any personal identifiers. The methods were conducted in accordance with the approved guidelines. All participants were provided with information about the purpose and procedures of the study and provided written informed consent before inclusion.

## Data Availability

The datasets generated during and/or analyzed during the current study are available from the corresponding author on reasonable request. The study protocol is available from www.clinicaltrials.gov.
